# The brain‐penetrant ATM inhibitor, AZD1390, promotes axon regeneration and functional recovery in preclinical models of spinal cord injury

**DOI:** 10.1002/ctm2.962

**Published:** 2022-07-12

**Authors:** Zubair Ahmed, Richard I. Tuxworth

**Affiliations:** ^1^ Neuroscience and Ophthalmology, Institute of Inflammation and Ageing University of Birmingham Birmingham UK; ^2^ Centre for Trauma Sciences Research University of Birmingham Birmingham UK; ^3^ Birmingham Centre for Genome Biology, Institute of Cancer and Genomic Sciences University of Birmingham Birmingham UK

Dear Editor,

This study demonstrates that AZD1390, an orally bioavailable, brain‐penetrant, potent, and highly selective inhibitor of ataxia–telangiectasia mutated (ATM), promotes dramatic recovery after spinal cord injury (SCI). AZD1390 engaged and suppressed its target and promoted dorsal root ganglion neuron (DRGN) neurite outgrowth in vitro and stimulated axon regeneration after SCI in vivo, with the recovery of conductance across the lesion site and led to remarkable improvements in sensory and locomotor function. AZD1390 is currently in clinical development for cancers.^1^ The simple oral administration route and good safety profile suggests that AZD1390 is a potential new therapy to promote recovery of function after SCI in patients.

SCI is a debilitating condition that affects between 200 000 and 1.2 million people every year.^2^ At present, there are no cures. Treating SCI is complicated and is hampered by the low intrinsic capacity of central nervous system (CNS) neurons to re‐grow after injury and the presence of extrinsic factors such as myelin‐ and scar‐derived inhibitory molecules in the environment of the regenerating axon.^3^ One cellular feature of SCI is the accumulation of unrepaired double‐strand breaks (DSBs) in DNA in neurons. DSBs lead to genome instability in replicating cells and can trigger apoptosis.^4^ In the CNS, they are potentially even more genotoxic as postmitotic neurons cannot be readily replaced, and the persistent activation of the DNA damage response is damaging.

DSBs are sensed by the MRN complex, composed of Mre11, Rad50, and NBS1/Nbn, leading to the activation of downstream ATM or ataxia–telangiectasia and Rad3‐related (ATR) kinases.^5^ ATM and ATR mediate many of the downstream events of the DNA damage response, such as cell‐cycle arrest, repair, and apoptosis. ATM is active in all stages of the cell cycle and regulates DSB repair via non‐homologous end‐joining and homologous recombination (HR). In contrast, ATR primarily regulates HR and is important for the resolution of replication stress. As HR is restricted to the S2 and G2 phases of the cell cycle, ATM is likely to be the principal regulator of DSB repair in postmitotic neurons.^5^, ^6^

We used an adult mouse DRGN culture system with added CNS myelin extracts, containing high titres of Nogo‐A, myelin‐associated glycoprotein, and chondroitin sulphate proteoglycans^7^ to mimic the environment of the injured CNS to test the effects of AZD1390 on DRGN survival and neurite outgrowth. The treatment of DRGN cultures with increasing concentrations of AZD1390 from 1 to 10 nM significantly reduced the levels of ATM activation (phosphorylated ATM [pATM] levels), with 5 nM being the lowest most effective dose (Figure 1A,B). Suppression of pATM with 5‐nM AZD1390 corresponded to a 60%, 88%, and 81% increase in DRGN survival, % DRGN with neurites, and longest neurite length compared to vehicle‐treated DRGN, respectively (Figure 1C–E). In comparison, another tool inhibitor of ATM, KU‐60019,^8^ showed similar levels of DRGN survival, % DRGN with neurites, and the mean longest neurite length as observed with AZD1390 (Figure 2C–F). These results suggest that the inhibition of ATM in vitro using either AZD1390 or KU‐60019 promotes similar levels of significant DRGN survival and disinhibited neurite outgrowth.

**FIGURE 1:**
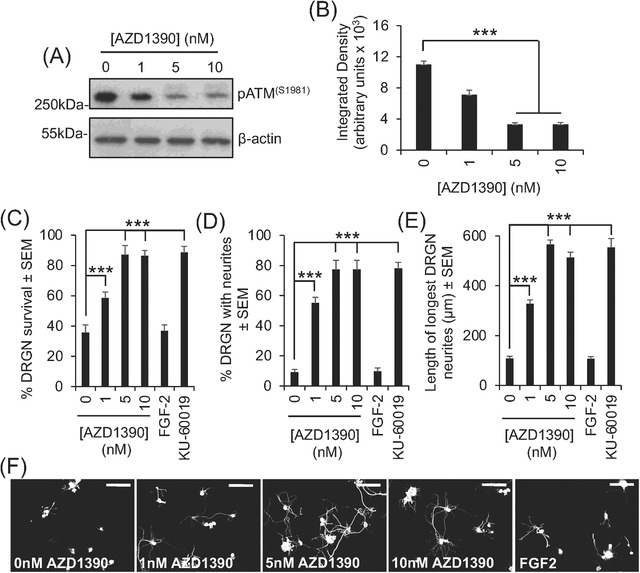
AZD1390 suppresses phosphorylated ataxia–telangiectasia mutated (pATM) levels and promotes significant dorsal root ganglion neuron (DRGN) neurite outgrowth in the presence of inhibitory central nervous system (CNS) myelin extracts. (A) Western blot and (B) densitometry to show that increasing concentrations of AZD1390 suppress pATM levels. Suppression of pATM promotes (C) DRGN survival, (D) % DRGN with neurites, and (E) the mean longest DRGN neurite length despite the presence of inhibitory concentrations of CNS myelin extracts. (F) Representative photomicrographs to show neurite outgrowth from each condition. Fibroblast growth factor‐2 (FGF2) was used as a positive control. KU‐60019 also promoted similar levels of DRGN survival and neurite outgrowth as AZD1390. Scale bars = 50 μm in (F). *n* = 3 wells/condition, three independent repeats (total *n* = 9 wells/condition). ***p < .0001, one‐way ANOVA with Dunnett's post hoc test

**FIGURE 2:**
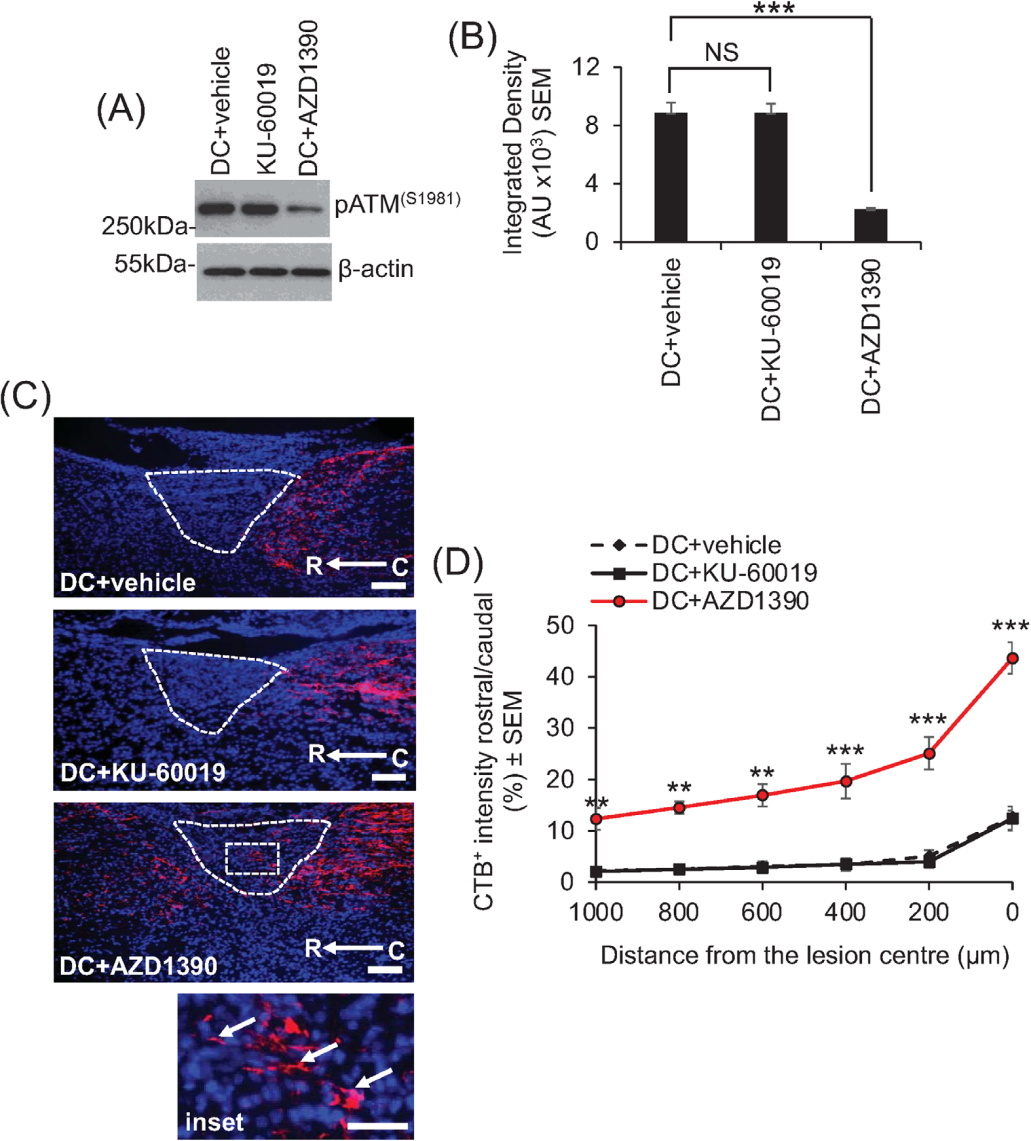
Suppression of phosphorylated ataxia–telangiectasia mutated (pATM) with oral AZD1390 promotes dorsal column (DC) axon regeneration in mice. (A) Western blot and (B) densitometry to show that oral AZD1390 (20 mg/kg, once daily) significantly suppresses pATM levels in dorsal root ganglia (DRG) at 4 weeks after DC injury. KU‐60019 (20 mg/kg, once daily), on the other hand, did not alter pATM levels by oral delivery. (C) Cholera toxin B (CTB)^+^ axons (red; nuclei = blue [stained with DAPI]) and (D) quantification to show that only oral AZD1390 treatment promoted axon regeneration through the lesion site and entry into the rostral cord. Fewer axons were present in the caudal cord in DC + vehicle and DC + oral KU‐60019‐treated animals and axons terminated at the lesion edge of the caudal cord. Inset in (C) shows high power magnification of the boxed region indicating CTB^+^ axons (arrows) growing through the lesion site towards the rostral cord. Dotted white lines show the injury site. C–R = caudal to rostral. Scale bars = 200 μm in (C). *n* = 6 mice/condition, two independent repeats (total *n* = 12 mice/group). **p < .001; ***p < .0001, one‐way ANOVA with Dunnett's post hoc test

After a dorsal column (DC) SCI in mice,^9^ only oral delivery of a previously optimized 20‐mg/kg dose of AZD1390, once daily,^1^ caused significant inhibition of ATM. Oral delivery of KU‐60019 had no effect on ATM levels, even at much higher levels up to 100 mg/kg (Figures 2A,B and S1). However, in separate experiments, intrathecal delivery of 10 µg of KU‐60019 after SCI suppressed pATM levels by 81% (Figure S1), consistent with our previously published report^8^ demonstrating that KU‐60019 is not available in the CNS after oral delivery but is available after intrathecal injection. Consistent with the suppression of pATM levels in AZD1390‐treated animals, cholera toxin B tracing of axons showed that only oral AZD1390, but not KU‐60019, promoted DC axon regeneration through the lesion site and into the rostral cord (Figure 2C,D).

Electrophysiology demonstrated that the normal compound action potential (CAP) trace across the lesion site was ablated after injury (Figure 3A). Only oral AZD1390 treatment, and not equivalent doses of oral KU‐60019, returned a significant CAP trace (Figure 3A), CAP amplitude (Figure 3B), and CAP area (Figure 3C). This return of electrophysiological function in AZD1390‐treated animals after SCI, together with the possible remodelling of local circuitry, correlated with significant improvements in sensory (Figure 3D) and locomotor (Figure 3E) function, such that AZD1390‐treated animals were indistinguishable from sham‐treated control animals by 4 weeks after SCI (Figure 3D,E).

**FIGURE 3:**
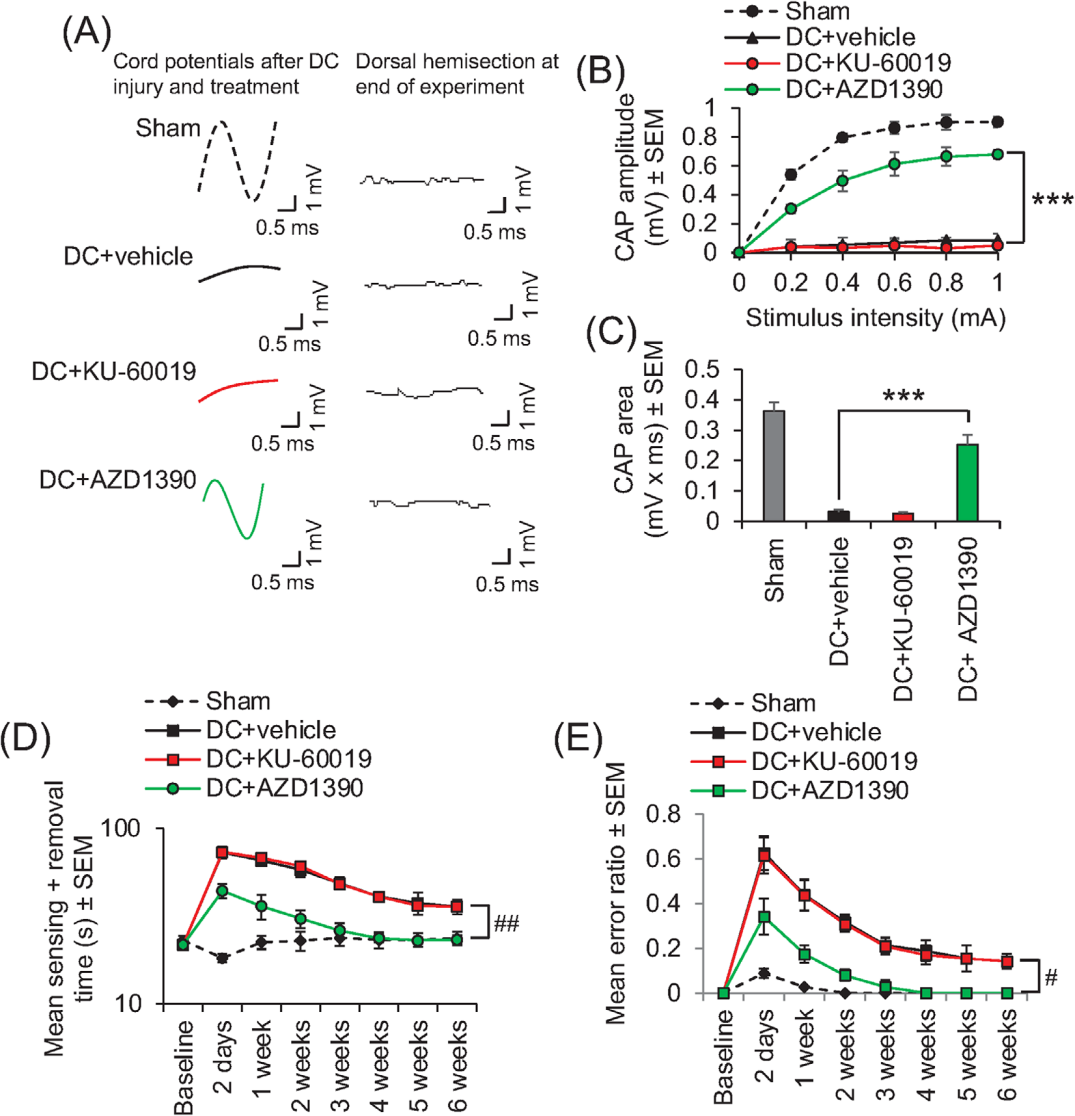
Suppression of phosphorylated ataxia–telangiectasia mutated (pATM) with AZD1390 promotes electrophysiological, sensory, and locomotor function recovery after dorsal column (DC) injury in mice. (A) Representative Spike 2 processed compound action potential (CAP) traces after oral delivery of ataxia–telangiectasia mutated (ATM) inhibitors. The normal CAP trace is ablated after DC injury and significantly restored by oral AZD1390 but not by oral KU‐60019 (both dosed at 20 mg/kg, once daily). Dorsal hemisection at the end of the experiments ablated all CAP traces demonstrating the technical success of the experiment. Oral AZD1390 significantly improved (B) CAP amplitudes and (C) CAP areas at 6 weeks after DC injury. Only oral AZD1390 significantly improved (D) tape sensing and removal times and (E) ladder crossing performance (locomotor function) over 6 weeks after DC injury. *n* = 6 mice/group, two independent experiments (total *n* = 12 mice/group). ***p = .0001, one‐way ANOVA with Dunnett's post hoc test. #*p* = .00011, linear mixed models; ##*p* = .00012, generalized linear mixed models

As the rat SCI model is considered to be a good translational model that accurately models the human SCI pathophysiology,^9^, ^10^ we also showed that oral AZD1390 and not oral KU‐60019 (both dosed at 20 mg/kg, once daily) suppressed ATM activation (Figure 4A,B) and could return a similar significant CAP trace (Figure 4C), CAP amplitude (Figure 4D), and CAP area (Figure 4E) after DC injury in adult rats, correlating with significantly improved sensory (Figure 4F) and locomotor (Figure 4G) function. We have previously shown that intrathecal delivery of KU‐60019 after DC injury in rats does engage its target and promotes significant electrophysiological, sensory, and locomotor improvements,^8^ but here, oral delivery of KU‐60019 had no effect again suggesting that orally delivered KU‐60019 does not reach the CNS.

**FIGURE 4:**
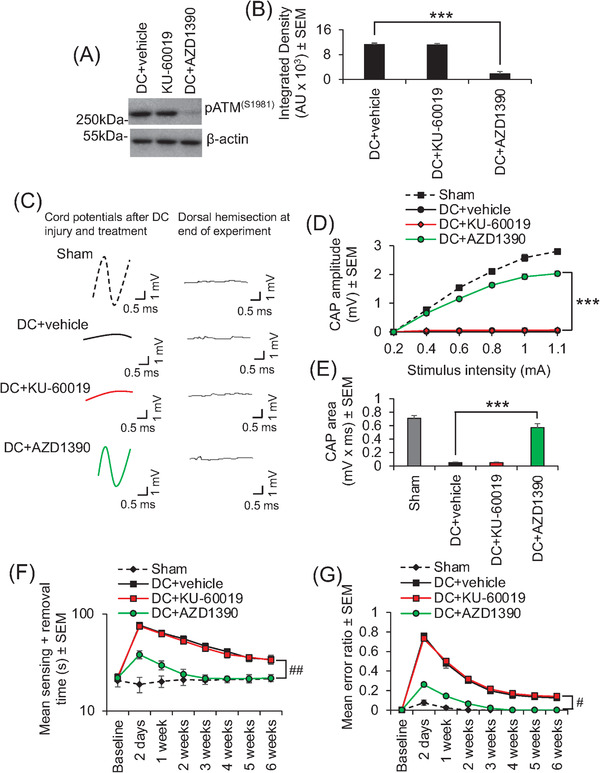
Suppression of phosphorylated ataxia–telangiectasia mutated (pATM) with AZD1390 also promotes electrophysiological, sensory, and locomotor function recovery after dorsal column (DC) injury in the rat. (A) Western blot and (B) densitometry show that oral AZD1390 (20 mg/kg, once daily) significantly suppresses pATM levels in dorsal root ganglia (DRG) at 4 weeks after DC injury in the rat. Again, KU‐60019 (20 mg/kg, once daily) did not alter pATM levels by oral delivery in rats. (C) Representative Spike 2 processed compound action potential (CAP) traces after oral delivery of ataxia–telangiectasia mutated (ATM) inhibitors. The normal CAP trace is ablated after DC injury and significantly restored by oral AZD1390 but not by oral KU‐60019 (both dosed at 20 mg/kg, once daily). Dorsal hemisection at the end of the experiments ablated all CAP traces demonstrating the technical success of the experiment. Oral AZD1390 significantly improved (D) CAP amplitudes and (E) CAP areas at 6 weeks after DC injury. Only oral AZD1390 significantly improved (F) tape sensing and removal times and (G) ladder crossing performance (locomotor function) over 6 weeks after DC injury. *n* = 6 rats/group, two independent experiments (total *n* = 12 rats/group). ***p = .0001, one‐way ANOVA with Dunnett's post hoc test. #*p* = .00011, linear mixed models; ##*p* = .00012, generalized linear mixed models

In conclusion, we show that brain‐penetrant, orally bioavailable AZD1390, engaged with its target in the CNS and promoted DRGN survival and neurite outgrowth in vitro and axon regeneration and functional recovery after SCI in both mice and rats in vivo. Therefore, AZD1390 could be a first‐in‐class reparative drug for the treatment of SCI with a favourable delivery mode.

## CONFLICT OF INTEREST

ZA and RIT are inventors on a patent related to this work.

## Supporting information

Figure S1 Intrathecal but not oral KU‐60019 suppresses pATM

## References

[1] Durant ST , Zheng L , Wang Y , et al. The brain‐penetrant clinical ATM inhibitor AZD1390 radiosensitizes and improves survival of preclinical brain tumor models. Sci Adv. 2018;4(6):eaat1719. 29938225 10.1126/sciadv.aat1719 PMC6010333

[2] Kang Y , Ding H , Zhou H , et al. Epidemiology of worldwide spinal cord injury: a literature review. J Neurorestoratol. 2018;2018:61–69.

[3] Fawcett JW . The struggle to make CNS axons regenerate: why has it been so difficult?. Neurochem Res. 2020;45(1):144–158. 31388931 10.1007/s11064-019-02844-y PMC6942574

[4] Brochier C , Langley B . Chromatin modifications associated with DNA double‐strand breaks repair as potential targets for neurological diseases. Neurotherapeutics. 2013;10(4):817–830. 24072514 10.1007/s13311-013-0210-9 PMC3805873

[5] Shiloh Y , Ziv Y . The ATM protein kinase: regulating the cellular response to genotoxic stress, and more. Nat Rev Mol Cell Biol. 2013;14(4):197–210. 23847781

[6] Lamarche BJ , Orazio NI , Weitzman MD . The MRN complex in double‐strand break repair and telomere maintenance. FEBS Lett. 2010;584(17):3682–3695. 20655309 10.1016/j.febslet.2010.07.029 PMC2946096

[7] Ahmed Z , Dent RG , Leadbeater WE , Smith C , Berry M , Logan A . Matrix metalloproteases: degradation of the inhibitory environment of the transected optic nerve and the scar by regenerating axons. Mol Cell Neurosci. 2005;28(1):64–78. 15607942 10.1016/j.mcn.2004.08.013

[8] Tuxworth RIT , Taylor MJ , Anduaga A‐M , et al. Attenuating the DNA damage response to double‐strand breaks restores function in models of CNS neurodegeneration. Brain Commun. 2019;1(1):fcz005. 32954257 10.1093/braincomms/fcz005 PMC7425387

[9] Surey S , Berry M , Logan A , Bicknell R , Ahmed Z . Differential cavitation, angiogenesis and wound‐healing responses in injured mouse and rat spinal cords. Neuroscience. 2014;275:62–80. 24929066 10.1016/j.neuroscience.2014.06.003

[10] Bazley FA , Hu C , Maybhate A , et al. Electrophysiological evaluation of sensory and motor pathways after incomplete unilateral spinal cord contusion. J Neurosurg Spine. 2012;16(4):414–423. 22303873 10.3171/2012.1.SPINE11684

