# Supplementary material for: The brain‐penetrant ATM inhibitor, AZD1390, promotes axon regeneration and functional recovery in preclinical models of spinal cord injury
Source: Clin Transl Med. 2022 Jul 12;12(7):e962. doi: 10.1002/ctm2.962 (PMC9274214; doi:10.1002/ctm2.962)

**SUPPLEMENTARY INFORMATION FILE**

**The brain penetrant ATM inhibitor, AZD1390, promotes axon regeneration and functional recovery in preclinical models of spinal cord injury**

**Short title:** AZD1390 promotes recovery after SCI

Zubair Ahmed^1,2,^* and Richard I. Tuxworth^2,3,^*

***Corresponding authors:** z.ahmed.1@bham.ac.uk (Z.A.); r.i.tuxworth@bham.ac.uk (R.I.T.).

**MATERIALS AND METHODS**

**Experimental Design**

The aim of this study was to determine the role of ATM inhibition using a brain penetrant ATM inhibitor, AZD1390, on recovery after spinal cord injury. Cell culture experiments using primary adult mouse DRGN were subjected to ATM inhibitors including AZD1390 and KU-60019 and DRGN survival and neurite outgrowth was assessed in the presence of inhibitory concentration of CNS myelin extracts to mimic the post-injury environment of the spinal cord. All experiments were performed with the investigator masked to the treatment conditions and unmasked after analysis. We then used our well-characterized *in vivo* dorsal column (DC) injury model of SCI in mice to determine the effect of ATM inhibition on axon regeneration and functional recovery. Since rat models of SCI are considered more translational than mouse, the *in vivo* DC injury model in rats was used to confirm that AZD1390 also promoted functional recovery after SCI. *In vivo* sample sizes were determined at the outset and based on power calculations derived from previous similar experiments in our laboratories. Animals were then randomly assigned to treatment groups roughly containing equal numbers of male and female mice and experimenters were masked to the treatment and procedural conditions. No animals were excluded for any reason and no expected or unexcepted adverse events were encountered. All animals tissue samples were processed at the same time and analyzed to prevent batch effects. Animals were housed in groups of four animals/cage in the same facility and the number of biological replicates is indicated in the figure legends. All measured outcomes were conducted by experimenters masked to the treatment conditions and unmasked after data analysis was complete.

**Animals**

All animal experiments were licensed by the UK Home Office and approved by the University of Birmingham’s Animal Welfare and Ethical Review Board. Surgical procedures were carried out accordance to the guidelines of the UK Animals Scientific Procedures Act, 1986, the Revised European Directive 1010/63/EU and conformed to the guidelines and recommendation of the use of animals by the Federation of the European Laboratory Animal Science Associations (FELASA). Experiments also conformed to the Animal Research: Reporting of *in vivo* Experiments (ARRIVE) guidelines. Adult male and female 6-8-week-old C57BL6 mice weighing 20-25g or 6-8-week-old adult Sprague-Dawley rats, weighing between 180-220g (all from Charles River, Margate, UK) were used in all experiments. Animals were housed in a standard animal facility maintained at 21^o^C and operating a 12-hour light-dark cycle, with free access to food and water. After surgery, animals were returned to their home cages and pre- and post-operative analgesia was provided as standard and as recommended by the named veterinary surgeon.

**Primary adult mouse DRGN cultures**

Primary adult mouse DRGN cultures were prepared as described by us previously ^1^. DRGN were dissociated using collagenase and cultured in supplemented Neurobasal-A (#10888022; NBA) containing B27 supplement (#17504044), L-glutamine (#25030081) and gentamicin (#15710064) (all from Invitrogen, Paisley, UK) at a plating density of 500/well in 8-well chamber slides (#C6932; Beckton Dickinson, Oxford, UK) pre-coated with 100μg/ml poly-D-lysine (#P6407; Sigma, Poole, UK). Glial cell proliferation was inhibited in cultures using 5-fluoro-2-deoxyuridine (5-FDU; #343333; Sigma) at 30µM ^1^. To mimic the inhibitory environment of the CNS, myelin extracts (CME; CNS myelin extracts) were prepared from adult C57BL6 mice as described by us previously ^1^, confirmed to contain significant MBP, Nogo-A, MAG and Brevican and used at 200 µg/ml to completely inhibit DRGN neurite outgrowth ^1^. Positive controls included pre-optimized fibroblast growth factor-2 (FGF-2) (#100-18C, Peprotech, London, UK; 10 ng/ml ^1^). Cells were cultured for 4 days in a humidified chamber at 37 °C and 5% CO2 before being subjected to immunocytochemistry, as described below. Treatments were added in triplicate and repeated on 3 independent occasions (i.e. total *n* = 9 wells/treatment) by an investigator masked to the treatment conditions.

**ATM inhibitors**

AZD1390 (gift from AstraZeneca) and KU-60019 (#4176; Tocris, Oxford, UK) were dissolved in DMSO (Sigma, Poole, UK) at stock concentrations of 10 mM for *in vitro* use. Stock concentrations were then diluted to the appropriate concentrations for *in vitro* use in supplemented NBA. For *in vivo* oral delivery, pre-optimized AZD1390 ^2^ and KU-60019 were dosed at 20mg/kg in 0.5% (w/v) hydroxypropyl methylcellulose (HPMC) and 0.1% (w/v) Tween 80, once daily by oral gavage. For intrathecal delivery of KU-60019 (10µg, every 24 hours) ^3^ stocks were diluted to the final doses in phosphate buffered saline.

**Immunocytochemistry and quantification of DRGN survival and neurite outgrowth**

DRGN were fixed in 8-well chamber slides *in situ* using 4% paraformaldehyde before being subjected to immunocytochemistry with rabbit anti-βIII tubulin antibodies (#T3952; 1:200 dilution; Sigma) to detect DRGN soma and neurites, as described previously ^1^. Alexa-488 goat anti-mouse IgG (#A32723; 1:400 dilution; ThermoFisher, Leicester, UK) secondary antibodies were used to visualize DRGN soma and neurites using an Axioplan 2 epifluorescent microscope equipped with an AxioCam HRc and running Axiovision Software (all from Carl Zeiss, Hertfordshire, UK).

With the investigator masked to the treatment conditions, the proportion of βIII-tubulin^+^ DRGN, the number of DRGN with neurites and the longest DRGN neurite length were all calculated using Axiovision Software, as described by us previously ^1^.

**Dorsal column crush injury model of SCI in mouse and rat**

Experiments comprised *n* = 6 animals/group: (1), Sham uninjured control group (anaesthesia followed by partial laminectomy but no DC crush injury); (2), DC crush injury + oral vehicle solution (0.5% HPMC and 0.1% Tween 80); (3), DC crush injury + oral AZD1390; and (4), DC crush injury + oral KU-60019. A final group of animals received intrathecal injection of KU-60019 every 24 hours through a cannula implanted into the subarachnoid space ^3^. Experiments were repeated on at least 2 independent occasions (i.e total *n* = 12 animals/group).

Animals were subcutaneously injected with Buprenorphine and anaesthetized using 5% isoflurane and 1.8 l/min O_2_. After a partial laminectomy at T8, DC were crushed bilaterally using calibrated watchmaker’s forceps as described by us previously ^4^. Animals were dosed immediately after injury and allowed to recover in their home cage. Drugs were given every 24 hours for the duration of each experiment.

For western blot, animals were killed at 4 weeks and tissues harvested as described below. For immunohistochemistry, electrophysiology and behavioural analysis, animals were treated up to 6 weeks after DC injury.

**Cholera toxin B labelling and quantification of DC regenerating axons**

For retrograde tracing of regenerating DC axons, 1% Cholera toxin B (CTB; #104, List Biologicals, Campbell, CA, USA) was injected into the sciatic nerve at mid-thigh levels, using glass microneedles, 1 week before killing mice with rising concentrations of CO_2_. Animals were intracardially perfused with 4% paraformaldehyde and CTB labelled axons were detected by immunohistochemistry using a goat polyclonal anti-CTB antibody (#703; 1:1000 dilution, List Biological Labs) at 6 weeks after injury, as described later.

CTB^+^ regenerating axons were quantified in sagittal sections of the spinal cord from the whole series of tissues for each mouse (total *n* = 12 mice/group). CTB intensity was quantified in ImageJ software (www.imagej.nih.gov) at different distance rostral to the injury center and expressed as a % of CTB intensity caudal to the injury site, which controls for slight variations in tracing efficiency.

**Tissue harvesting**

Methods for tissue harvesting have been described by us previously ^1,3^. Briefly, for western blot analysis: animals were killed by rising concentrations of CO_2_ and L4/L5 DRG pairs were dissected out and snap frozen in liquid nitrogen and stored at -80 ^o^C until required ^5^. For immunohistochemistry: animals were killed in rising concentrations of CO_2_ and intracardially perfused with 4% paraformaldehyde ^3,5^. The SCI site + 5 mm either side of the lesion center was dissected out and postfixed in 4% formaldehyde for 2 hr, followed by cryoprotection in a graded series of sucrose and embedded in optimal temperature cutting medium (#12678646, ThermoFisher). Sections were cut at 15 µm-thick and stored at -80^o^C until required.

**Western blot analysis and densitometry**

Total protein was extracted from DRGN cultures and fresh DRG tissues as described by us previously ^1,3^. To obtain enough protein to run western blots, total protein from 3 separate wells/condition were pooled together whilst L4/L5 DRG pairs were combined from 2 mice/group. Western blots were then repeated on at least 3 independent occasions. Briefly, cultures/DRG tissues were washed in PBS and total protein extracted in ice-cold lysis buffer containing 20 mM HEPES, 1 mM EDTA, 150 mM NaCl, 1% NP-40 and 1 mM DTT and supplemented with protease (#P8340) and phosphatase inhibitor cocktails (#P5726) (all from Sigma). After a protein assay using the Bradford Assay Kit (#5000001; BioRad, Watford, UK), 15 µg of total protein was separated on 12% Tris-glycine SDS-PAGE gels. Proteins were then transferred onto PVDF membranes (#IEVH00005, Merck, Gillingham, UK), blocked in non-fat milk and incubated with rabbit anti-pATM (Ser1981) antibody (#13050, 1:200 dilution, Cell Signalling Technology, London, UK) overnight at 4^o^C. Membranes were then washed in Tris buffered saline (TBS) and incubated with appropriate mouse/rabbit HRP-labelled secondary antibody (#GENA934; 1:1000 dilution, GE Healthcare, Buckinghamshire, UK) and bands detected using an enhanced chemiluminescence kit (#RPN2108, GE Healthcare). Rabbit anti-β-actin antibody (#ZRB1312, 1:1000; Sigma) was used as a loading control for western blots.

For densitometry, western blots were scanned into Adobe Photoshop (Adobe Systems, San Jose, CA, USA) keeping all scanning parameters the same between each blot and densitometrically analysed using the built-in gel plotting macros in ImageJ (ww.nihimage.nih.gov), as previously described ^1^.

**Immunohistochemistry**

Sections were thawed at room temperature and after washes in PBS, permeabilized in PBS containing 0.1% Triton X-100, blocked in PBS containing 3% bovine serum albumin and 0.05% Tween-20 (all from Sigma) and incubated in primary antibodies, as described by us previously ^3^.

**Electrophysiology**

Compound action potentials (CAP) were recorded at 6 weeks after DC injury and treatment as described by us previously ^3,6,7^. Briefly, with the experimenter masked to the treatment conditions, CAPs across the surface of the lesion site were recorded using silver wire electrodes stimulated at lumbar (L1)-L2 and CAPs recorded at cervical (C)4-C5. Spike 2 software (Cambridge Electronic Design, Cambridge, UK) was used to process and analyse the traces and determine the CAP amplitude and CAP area for each condition.

**Functional tests after DC injury and treatment**

Tape sensing and removal and horizontal ladder crossing tests were used to detect sensory and locomotor changes after DC injury and treatment as described by us previously ^3,6,7^. Briefly, tape sensing and removal was tested by attaching a 15x15 mm piece of sticky tape (Kip Hochkrepp, Bocholt, Germany) onto the plantar surface of each the left paw of each mouse and recording the time taken to detect and remove the tape. For the ladder crossing test, each mouse was trained prior to DC injury to master the traversing the ladder (0.9 m long and 15.5 cm wide with randomly adjusted rungs). Animals were assessed crossing the ladder and the total number of steps and the number of left and right hind paw slips were recorded and presented as an error ratio.

All of these tests were performed at 2 days after injury, then at 1 week followed by weekly tests for 6 weeks. The experimenter was masked to the treatment conditions and animals were assessed in the same order and time of day with each test performed for 3 individual trials on each occasion.

**Statistical analysis**

All results are presented as mean ± standard error of the mean (SEM). Statistical significance was calculated by one-way analysis of variance (ANOVA) with post-hoc Dunnett’s method using SPSS Statistics 19 (IBM, New York, USA). For the horizontal ladder crossing and tape removal tests, data was analyzed as described previously ^3,6,7^ using R statistics package (www.r-project.org). Briefly, whole time-course of lesioned and sham-treated animals in the ladder crossing test was compared using binomial generalized linear mixed models (GLMM), fitted in R using *lme4* plugin with the *glmer* plugin function and *p* values calculated using parametric bootstrap. For the tape sensing and removal test, linear mixed models (LMM) were calculated by model comparison in R using the *pbkrtest* plugin, with the Kenward-Roger method.

**REFERENCES**

1. Ahmed Z, Dent RG, Suggate EL, et al. Disinhibition of neurotrophin-induced dorsal root ganglion cell neurite outgrowth on CNS myelin by siRNA-mediated knockdown of NgR, p75NTR and Rho-A. *Mol Cell Neurosci.* 2005;28(3):509-523.

2. Durant ST, Zheng L, Wang Y, et al. The brain-penetrant clinical ATM inhibitor AZD1390 radiosensitizes and improves survival of preclinical brain tumor models. *Sci Adv.* 2018;4(6):eaat1719.

3. Tuxworth RI, Taylor MJ, Martin Anduaga A, et al. Attenuating the DNA damage response to double-strand breaks restores function in models of CNS neurodegeneration. *Brain Commun.* 2019;1(1):fcz005.

4. Ahmed Z, Bansal D, Tizzard K, et al. Decorin blocks scarring and cystic cavitation in acute and induces scar dissolution in chronic spinal cord wounds. *Neurobiol Dis.* 2014;64:163-176.

5. Surey S, Berry M, Logan A, Bicknell R, Ahmed Z. Differential cavitation, angiogenesis and wound-healing responses in injured mouse and rat spinal cords. *Neuroscience.* 2014;275:62-80.

6. Almutiri S, Berry M, Logan A, Ahmed Z. Non-viral-mediated suppression of AMIGO3 promotes disinhibited NT3-mediated regeneration of spinal cord dorsal column axons. *Sci Rep.* 2018;8(1):10707.

7. Kitchen P, Salman MM, Halsey AM, et al. Targeting Aquaporin-4 Subcellular Localization to Treat Central Nervous System Edema. *Cell.* 2020;181(4):784-799 e719.


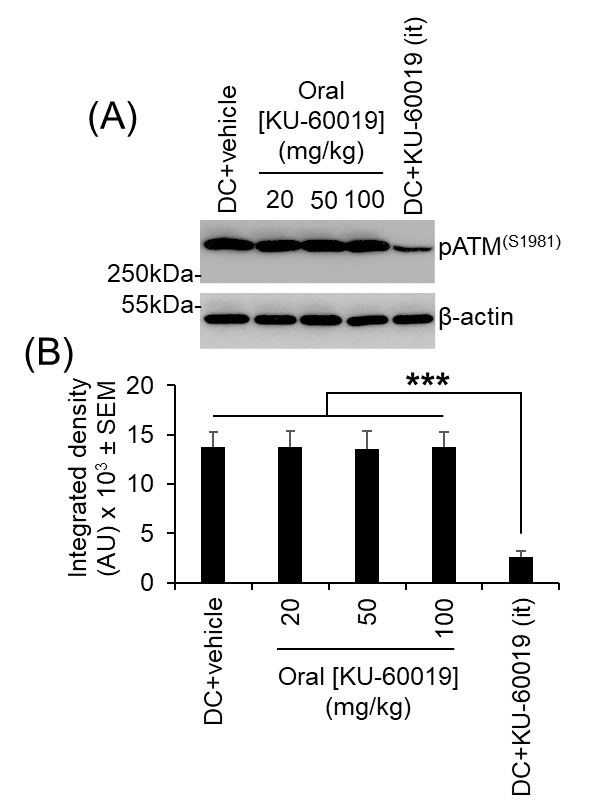


**Figure S1.** Intrathecal but not oral KU-60019 suppresses pATM. (A) Western blot and (B) densitometry to show that oral KU6000-19 had no effect on pATM levels but intrathecal delivery of a previously published concentration of KU-60019, promoted 81% inhibition of pATM.


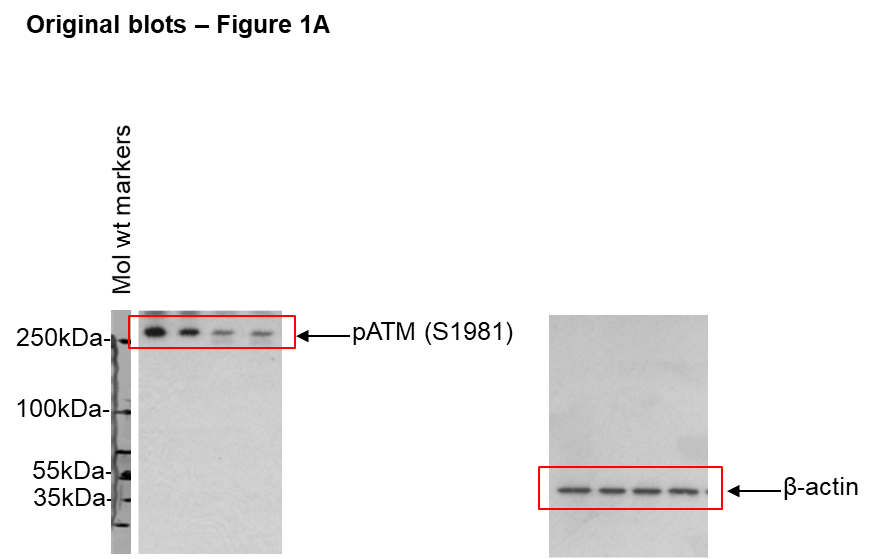


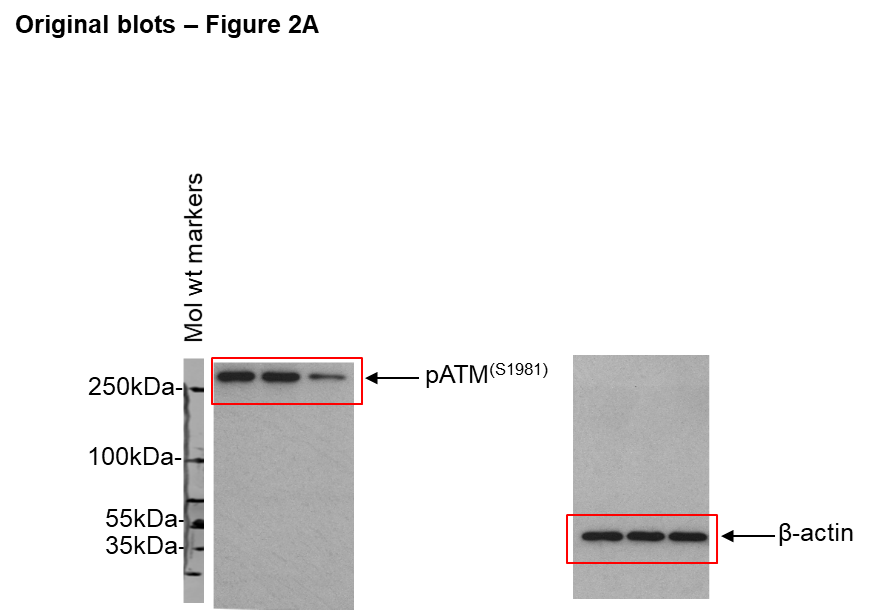


**Original blots - Figure 4A**

**
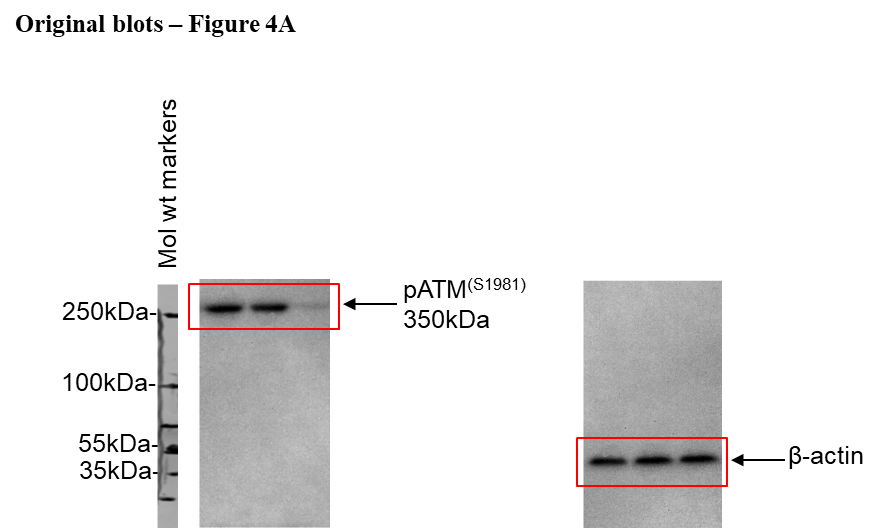
**

**Original blots - Figure S1A**


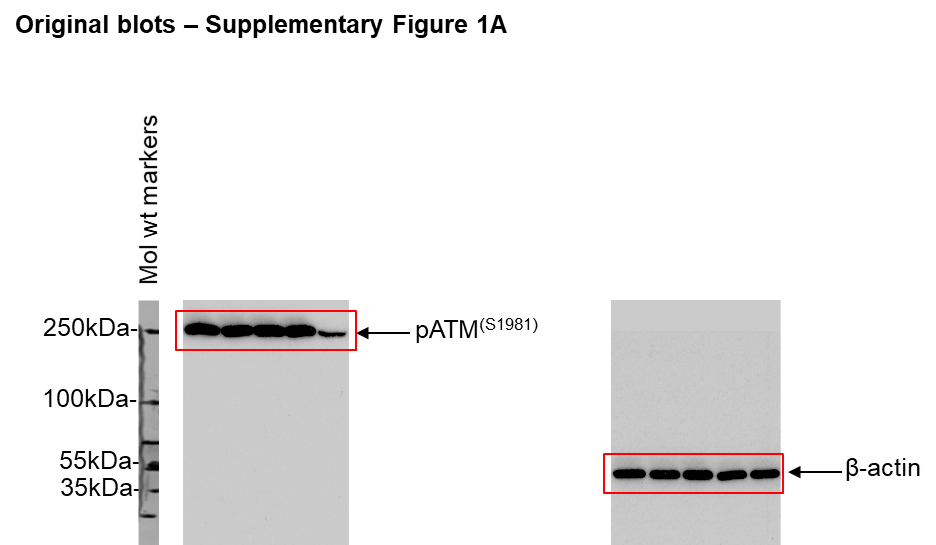

Supplement: Supplementary file 1 — Figure S1 Intrathecal but not oral KU‐60019 suppresses pATM [file CTM2-12-e962-s001.docx]
